# Dynamic NMDAR-mediated properties of place cells during the object place memory task

**DOI:** 10.3389/fnbeh.2013.00202

**Published:** 2013-12-17

**Authors:** Thomas W. Faust, Sergio Robbiati, Tomás S. Huerta, Patricio T. Huerta

**Affiliations:** ^1^Laboratory of Immune and Neural Networks, Feinstein Institute for Medical Research, North Shore LIJ Health SystemManhasset, NY, USA; ^2^Department of Molecular Medicine, Hofstra North Shore LIJ School of MedicineManhasset, NY, USA

**Keywords:** OPM task, object placement, object location memory, CPP, place field, spatial information, mouse

## Abstract

N-methyl-D-aspartate receptors (NMDAR) in the hippocampus participate in encoding and recalling the location of objects in the environment, but the ensemble mechanisms by which NMDARs mediate these processes have not been completely elucidated. To address this issue, we examined the firing patterns of place cells in the dorsal CA1 area of the hippocampus of mice (*n* = 7) that performed an object place memory (OPM) task, consisting of familiarization (T1), sample (T2), and choice (T3) trials, after systemic injection of 3-[(±)2-carboxypiperazin-4yl]propyl-1-phosphate (CPP), a specific NMDAR antagonist. Place cell properties under CPP (CPP–PCs) were compared to those after control saline injection (SAL–PCs) in the same mice. We analyzed place cells across the OPM task to determine whether they signaled the introduction or movement of objects by NMDAR-mediated changes of their spatial coding. On T2, when two objects were first introduced to a familiar chamber, CPP–PCs and SAL–PCs showed stable, vanishing or moving place fields in addition to changes in spatial information (SI). These metrics were comparable between groups. Remarkably, previously inactive CPP–PCs (with place fields emerging *de novo* on T2) had significantly weaker SI increases than SAL–PCs. On T3, when one object was moved, CPP–PCs showed reduced center-of-mass (COM) shift of their place fields. Indeed, a subset of SAL–PCs with large COM shifts (>7 cm) was largely absent in the CPP condition. Notably, for SAL–PCs that exhibited COM shifts, those initially close to the moving object followed the trajectory of the object, whereas those far from the object did the opposite. Our results strongly suggest that the SI changes and COM shifts of place fields that occur during the OPM task reflect key dynamic properties that are mediated by NMDARs and might be responsible for binding object identity with location.

## Introduction

The hippocampus plays a critical role in the rapid formation of spatial, temporal, associative and episodic memories. Seminal work revealed that patients with right temporal lobectomy (Smith and Milner, 1981, 1989; Nunn et al., 1998, 1999) or specific damage to the hippocampus (Vargha-Khadem et al., 1997; Bohbot et al., 1998; Stepankova et al., 2004), as well as hippocampectomized macaques (Parkinson et al., 1988), do not recognize a visual object that has been moved from a familiar to a novel location implicating the hippocampal formation in object place memory (OPM; also referred to as object location memory). In rodents, OPM has been investigated with an open field format in which animals explore two objects spontaneously (sample trial) and, after a variable delay, are re-exposed to the same objects, one of which has been moved to a new location (choice trial). The exploration bias for the displaced object is used to quantify OPM (Ennaceur et al., 1997; Dix and Aggleton, 1999). This task is sensitive to lesions in the hippocampus, fornix, anterior thalamus, cingulate cortex, postsubiculum, and parasubiculum (Ennaceur et al., 1997; Bussey et al., 2000; Warburton et al., 2000; Liu et al., 2001; Mumby et al., 2002; Barker and Warburton, 2011). Pharmacological evidence shows that of N-methyl-D-aspartate receptor (NMDAR) antagonists can disrupt OPM when given before the sample phase, either systemically (Larkin et al., 2008) or by injection into the dorsal hippocampus (Assini et al., 2009; Barker and Warburton, 2013) or postsubiculum (Bett et al., 2013). In the latter, blocking the α-amino-3-hydroxy-5-methyl-4-isoxazolepropionic acid receptor (AMPAR) is also effective (Bett et al., 2013). Notably, studies in rats show that NMDAR antagonism after the sample but before the choice phase (with delays of 1 or 24 h) does not alter OPM (Larkin et al., 2008; Warburton et al., 2013).

Studies of freely moving rodents with surgically implanted electrodes directed to the CA1 area of the hippocampus have shown that pyramidal neurons function as *place cells*, such that each of them displays enhanced spiking activity within a discrete area of the environment, called the cell's *place field* (O'Keefe and Dostrovsky, 1971; O'Keefe and Nadel, 1978; O'Keefe, 2007). When an animal explores a novel environment, place cells develop fields *de novo* and show increased spatial specificity after familiarization (Hill, 1978; Wilson and McNaughton, 1993; Frank et al., 2004). The spiking of place cells is strongly influenced by distal cues and large barriers within an arena, whereas local cues have a weak effect (Muller and Kubie, 1987; Cressant et al., 1997, 1999; Lever et al., 2002; Renaudineau et al., 2007). Nevertheless, local objects can modulate place cell firing (Lenck-Santini et al., 2005; Komorowski et al., 2009; Manns and Eichenbaum, 2009; Burke et al., 2011; Deshmukh and Knierim, 2013). For instance, place fields located near objects experience large remapping when the objects are rotated, compared to place fields far from objects (Lenck-Santini et al., 2005). Also, changing the configuration of multiple objects causes increased remapping of place fields in rats running on a linear track (Burke et al., 2011). A recent study in which rats are exposed to four local objects describes place cells that develop several place fields, such that each field is located at a similar distance and direction from a different object, leading the authors to call them *landmark vector* cells (Deshmukh and Knierim, 2013). Moreover, place cells can remap in the absence of environmental alterations, as shown by a study with rats subjected to contextual fear conditioning, which undergo greater CA1 place field remapping in the conditioning context as compared to a control environment (Moita et al., 2004). Furthermore, during place preference tasks and water maze tasks, place cells exhibit either increased firing or accumulation of place fields in goal locations (Hollup et al., 2001; Hok et al., 2007). Interestingly, the addition of place fields in goal locations depends on the activation NMDARs (Dupret et al., 2010).

NMDARs are abundantly located in glutamatergic synapses and are essential for the induction of synaptic plasticity, which is typically studied with the paradigm of long-term potentiation (LTP) [reviewed in Bliss et al. (2007)]. Mice with a genetic knockout (KO) of the obligatory NR1 subunit of the NMDAR in hippocampal pyramidal cells (Tsien et al., 1996) exhibit place cells with larger fields and decreased spatial specificity (McHugh et al., 1996; Tonegawa et al., 1996). Studies in rats have shown that NMDARs mediate the long-term stability of place cells following the animal's introduction to a novel environment, in a manner that requires physical rather than pure visual experience of the environment (Kentros et al., 1998; Rowland et al., 2011). NMDAR antagonism also prevents the increased in-field firing and the center-of-mass (COM) shifting of the place field, which is normally observed following multiple passes through a place field in a linear track (Ekstrom et al., 2001). An interesting study using a different paradigm than NMDAR antagonism has shown that induction of LTP can cause long-term remapping of place fields in a subset of place cells (Dragoi et al., 2003), bolstering the idea that changes in spatial coding in CA1 underlie hippocampal learning and memory.

During the OPM task, the initial location of objects and the subsequent movement of one of the objects might be encoded through changes in the spatial coding of place cells that might be mediated by NMDARs. Alternatively, OPM might be based on neural mechanisms that do not require changes in the spatial coding of place cells. Here, we present evidence that place cells in the dorsal CA1 area participate in encoding the location and movement of objects by changes in their spatial information (SI) and by shifts in their place fields, and these dynamic properties depend on the activation of NMDARs.

## Materials and methods

### Animals

The Feinstein Institute Animal Care and Use Committee approved all animal procedures. Female BALB/cJ mice (Jackson Labs) were chosen because they maintained high levels of object exploration throughout the task, thus optimizing data collection (Chang and Huerta, 2012). Mice were housed in groups of five per cage and maintained on a reverse schedule of 12 h of darkness (07:00 to 19:00) and 12 h of light, with *ad libitum* access to food and water. Starting 1 week before testing, mice were handled for 5 days in daily sessions of 5–10 min. Handling and subsequent experiments were conducted during the dark period of their circadian cycle. After surgery, mice were housed individually.

### OPM task

The apparatus consisted of a chamber with a square base (40 cm on the side) and 60-cm high walls built of polyvinyl chloride. Three walls were made opaque with black inserts, while the fourth wall was transparent. The floor was covered with black bedding that was similar in texture to the bedding used in the home cage except for the color. A light bulb (50 W) of orange–red hue illuminated the chamber from above. An infrared-sensitive camera (Panasonic) was mounted above the chamber and was connected to the video input of the behavioral tracking software (Ethovision XT8.5, Noldus) that tracked the animal's position during experimental trials. The software acquired the coordinates of the nose at 30 frames per s, which was facilitated by the high contrast between the animals' white coat at the black background.

**Figure 1:**
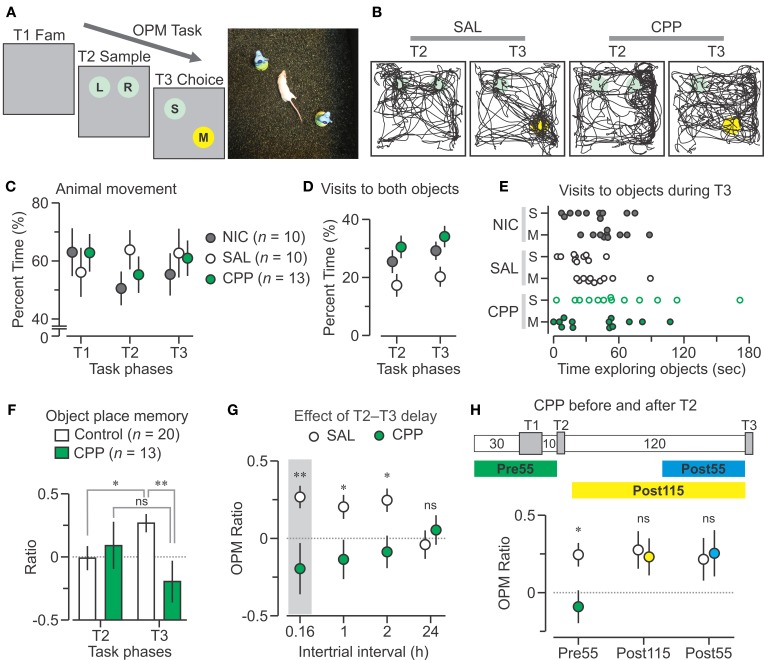
**CPP disrupts the OPM task. (A)** Left, schematic of the object place memory (OPM) protocol comprising a familiarization trial (T1 Fam., 15 min), sample (T2, 5 min), and choice (T3, 5 min), separated by 10-min delays. Right, top view of a mouse performing a choice trial within the square chamber (40 cm on the side). **(B)** Track plots of T2 and T3 for mice injected with saline (SAL, 0.9%) and CPP (10 mg per kg, 2.5 mg per mL in 0.9% saline). The SAL animal preferentially explores the moved object in T3, whereas the CPP mouse does not. Circles represent locations of the objects; colors, as in **(A)**. **(C)** Graph showing the percent time (mean ± s.e.m.) the animals spend moving during the trials; NIC, non-injected group. **(D)** Percent time (mean ± s.e.m.) of the combined visits to both objects during T2 and T3. **(E)** Graph for the time spent exploring the objects during T3 for each animal. **(F)** Discrimination ratios (mean ± s.e.m.) plotted across T2 and T3 showing that Control mice (SAL plus NIC) have a robust bias for the moved object on T3, whereas CCP mice do not. **(G)** OPM ratios (mean ± s.e.m.) for different delays between T2 and T3. The shaded area represents the T3 data from **(F)**. **(H)** *Top*, diagram of the time course of the task with injection of CPP 55 min before T2 (Pre55), 115 min before T3 (Post115) or 55 min before T3 (Post55). *Bottom*, OPM ratios (mean ± s.e.m.) for the CPP treatments. Abbreviations, L, left object; M, moved object; R, right object; S, static object; ns, non-significant; *, *P* < 0.005; **, *P* < 0.001 (*t*-test).

We tested naïve mice (*n* = 33), without microdrive implants, to validate our version of the OPM task. Animals were divided into two squads (*n* = 15 in squad 1, *n* = 18 in squad 2). They were transported inside their home cages into the darkened experimental room and placed in the empty chamber, one at a time, for four sessions (2 per day) of 15 min. On the third day, mice were divided into a CPP group (*n* = 5 in squad 1, *n* = 8 in squad 2) which received d-CPP injection (i.p. 10 mg per kg, Sigma Aldrich), a saline control group (SAL, *n* = 5 in squad 1, *n* = 5 in squad 2) which was injected with sterile saline solution (i.p. 0.9%), and a non-injected control group (NIC, *n* = 5 in squad 1, *n* = 5 in squad 2). The injections were timed so that each mouse was tested 30–45 min after being injected. The OPM task (Figure 1A) consisted of a familiarization trial (T1), a sample trial (T2), and a choice trial (T3), interspersed by 10-min delays that were spent in a highly habituated holding chamber. For T1, animals were placed in the empty chamber for 15 min. For T2, mice explored the chamber for 5 min in the presence of two identical objects, which were located in two of four possible sites at the center of the NW, NE, SW, or SE quadrants of the chamber, implying that each object was 10 cm from the two near walls and 30 cm from the distant walls. We used five pairs of objects of similar size (diameter at the base, 4.5 cm, height, 7–8 cm) that were glued to metal platforms so mice could not displace them. Object identity and starting location were chosen at random. For T3, which lasted 5 min, one object (chosen at random) remained in the familiar position while the second object was moved to a location that was the center of the adjacent quadrant, 20 cm apart from its previous position. We measured object exploration with a software algorithm (Ethovision) that assigned a circular zone (diameter, 6.5 cm) around each object and recorded the episodes in which the animal's snout was in close proximity (<1 cm) to the object's periphery. We have previously validated software-based methods for animal tracking (Huerta et al., 2000; Chang and Huerta, 2012). We used the number of visits and the times spent exploring each object on T2 and T3 for statistical comparisons. For T2, an exploration ratio was defined as “the time exploring the right object (RO) (in either NE or SE zone) divided by the sum of the times exploring both objects.” For T3, an OPM ratio was defined as “the time exploring the moved object minus the time exploring the stable object over the sum of the times exploring both objects.”

We used naïve mice (*n* = 32) to examine the effect of T2–T3 delays (1, 2, or 24 h) on OPM. As described above, animals were acclimated to the task for 2 days and, on day 3, received CPP or SAL injections (*n* = 16 per group) followed by the OPM task. This time, instead of 10 min, the T2–T3 delay was either 1 or 2 h; eight mice per group were tested for each delay. On day 6, the CPP group was retested, without being injected, with a 10-min delay. On day 15, all mice were injected again, tested on T2, returned to the colony, and tested on T3, 24 h later. Subsequently, we studied the effect of post-training CPP injections by using a T2–T3 delay of 2 h. One squad was injected with CPP (*n* = 8) or SAL (*n* = 8) 5 min after T2, with T3 occurring 115 min later. Another squad was injected with CPP (*n* = 8) or SAL (*n* = 8) 65 min after T2, with T3 occurring 55 min later.

### Electrode array implantation

We have described the construction of electrode arrays previously (Chang et al., 2013). Tetrodes were fabricated with polyimide-insulated wires (diameter, 17.8 μm) consisting of 90% platinum and 10% iridium (California Fine Wire, Grover Beach, CA). Tetrodes were wound using a small steel clamp and magnetic stir plate. Following roughly 30–40 rotations per cm wire length; the four wires were fused with a heat gun, threaded into a polyimide cannula array, and attached to a mobile microdrive block on a customized Plexiglas base. Tetrodes were pinned to the EIB-18 chip (Neuralynx, Bozeman, MT) and gold-plated down to a resistance of 50–150 kΩ on the day of implantation.

**Figure 2:**
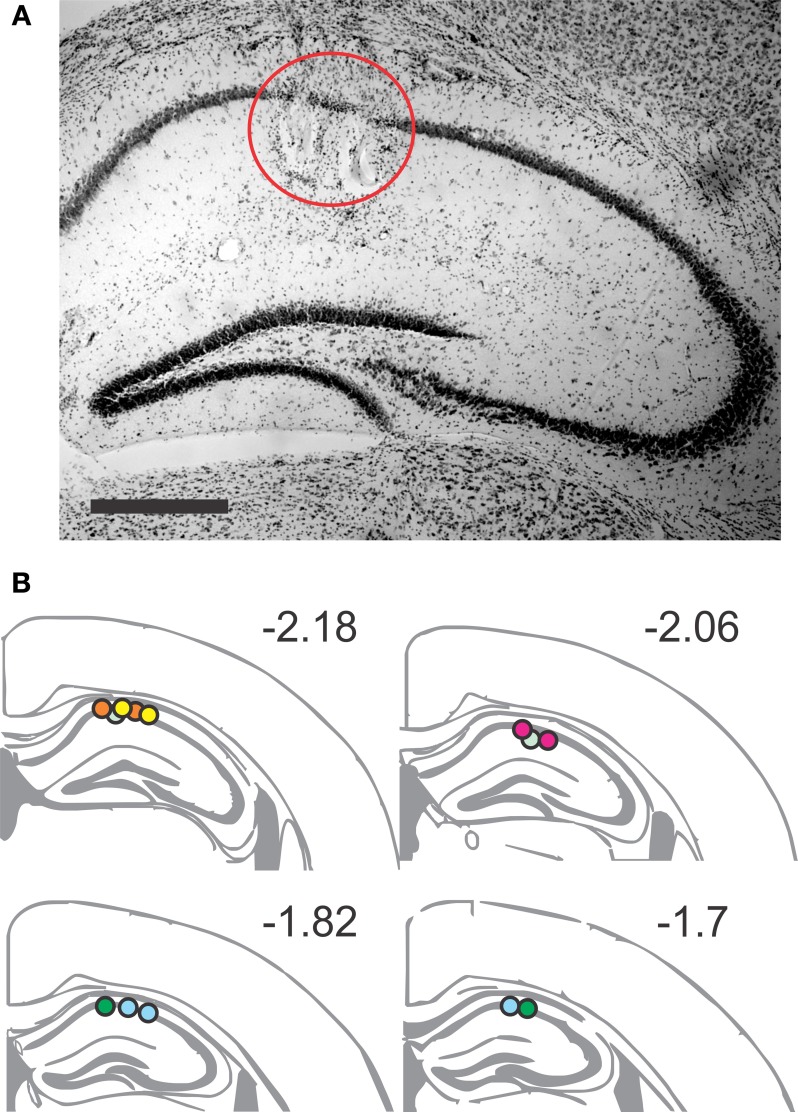
**Placement of recording sites in the dorsal CA1 area. (A)** Representative Nissl-stained section showing two lesions of the tetrode tips in the *stratum pyramidale*. Scale bar, 1 mm. **(B)** Coronal brain schematics, extending from AP −2.18 to AP −1.7, indicate the recording positions for the tetrodes. Arbitrary color code identifies the positions belonging to each animal, verified by lesion sites (*n* = 20).

A survival surgery was conducted on adult mice (3–6 mo of age), weighing 21–25 g. Animals were anaesthetized with 1.5–2.5% isoflurane, delivered with O_2_ (2 L per min), and were placed in a stereotaxic frame. Using a surgical drill (Foredom Electric, Bethel, CT), two craniotomies were made, one on the occipital bone for a brass ground screw and another above the parietal bone for the tetrode array. The *dura mater* was removed for the latter craniotomy. Mice were implanted with customized microdrives weighing ~1.5 g, including the dental cement. The array was targeted to the right dorsal CA1 area, using the coordinates of −2.0 mm AP, +1.6 mm ML from bregma, and lowered to an initial depth of 840 μm below the brain surface (Figure 2). The exposed craniotomy was filled with sterile Vaseline. As the dental acrylic hardened, mice were injected with buprenorphine (0.05 mg per kg) subcutaneously prior to removal of anesthesia. Mice were closely monitored for the first few hours post-surgery, and were monitored daily thereafter.

### Electrophysiological recordings

**Figure 3:**
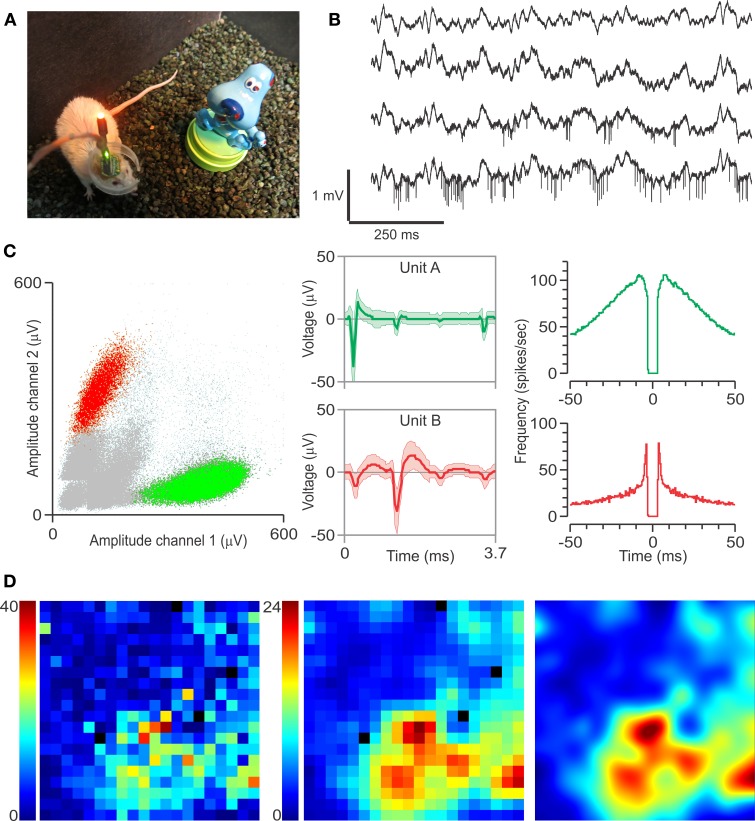
**Multi-electrode recordings, single units, and spatial firing in CA1. (A)** Implanted mouse with headstage attached, near one of the objects used in the study. **(B)** Local field potentials from four separate tetrodes, referencing a local reference electrode, with action potentials visible in the bottom two channels. **(C)** Left, clusters obtained from the bottom channel depicted in **(B)**. Middle, spike waveforms (mean ± *SD*) for the clustered single units. Right, autocorrelograms for these single units. The differences in spike waveforms and autocorrelograms show that Unit A is a putative interneuron, whereas Unit B is a putative pyramidal cell. **(D)** Representative rate map of unit B during the familiarization session. Color scale corresponds to frequency (spikes per second). Left, raw rate map of the arena divided into 2 cm^2^ pixels, with black pixels representing unexplored areas of the arena. Middle, the same rate map after smoothing with a 3-by-3 Gaussian kernel. Smoothed rate maps are used for subsequent analysis. Right, bicubic interpolation of the smoothed rate map is used for graphical presentation of place fields.

We recorded neural activity via a unitary gain headstage preamplifier (HS-18; Neuralynx Bozeman, MT), which was connected to a programmable amplifier (Lynx-8, Neuralynx) linked to the acquisition software (Cheetah 32, Neuralynx). Single units were recorded at a sampling rate of 30 kHz, band-pass filtered (600–6 kHz), and referenced to a nearby 50-μm local reference electrode in corpus callosum above dorsal CA1. Local field potentials were also acquired at a sampling rate of 3 kHz, band-pass filtered (0.1–6 kHz), and referenced to a ground screw above the cerebellum. The headstage also included one red-emitting LED and one green-emitting LED that were used for tracking the animal's position at 30 frames per s (Figure 3A). This was accomplished by linking an infrared-sensitive camera, mounted above the chamber, to the video input of the Cheetah software.

### OPM task in implanted mice

Three days post-surgery, the implanted mice were gradually habituated to the behavioral arena for a period of 3–5 days. Animals was transported inside the home cage into the darkened experimental room. Immediately after connecting the microdrive to the recording system, they were left to explore the empty chamber for sessions of 15–20 min, for a cumulative duration of over 1 h, to insure full acclimation to the context. During this time, the electrode array was advanced 35–140 μm per day until the tips reached their final recording depth at the level of *stratum pyramidale* of dorsal CA1 (Figure 2). This was confirmed by the presence of sharp waves and ripple events in the local field potential (O'Keefe, 1976; Buzsáki, 1986), and multiple units with high spike amplitude per tetrode. On days of behavioral testing, mice were injected (i.p.) with either d-CPP (10 mg per kg) or sterile SAL (0.9%) 30 min before testing began. The injections of CPP and SAL were alternated between days. As in naïve mice, the behavioral paradigm consisted of a 15-min T1 trial followed by a 10-min delay, a 5-min T2 trial followed by a 10-min delay, and a 5-min T3 trial. We measured object exploration by counting the number of visits to them. For each mouse, up to five pairs of objects of similar size were used, each only once. Object identity, starting location, and movement were randomized. Testing was conducted once daily until either all the objects had been presented, or until well-isolated units were no longer observed.

### Quantification of spiking properties

The neural signals were analyzed offline with a focus on single units and their spatial properties. We isolated single units manually using the clustering software KlustaKwik 1.5 (Kenneth Harris, http://klustakwik.sourceforge.net; Harris et al., 2000). Single units that contained a clear refractory period in their autocorrelograms were categorized as putative pyramidal cells or interneurons, with the latter not included in the subsequent analysis. For single units to be considered pyramidal cells, they needed to have a tendency to fire in bursts and a spike width (time between peak to trough) of ≥250 μ s (Resnik et al., 2012). Rate maps for arena occupancy and unit firing were computed with Neuroexplorer 3 (http://www.neuroexplorer.com), with the 40-cm^2^arena represented as a 20 × 20 matrix of 2-cm^2^ pixels. We constructed firing rate maps by calculating the total number of spikes for each pixel and then dividing by the dwell time for a particular session. Pixels in which the animal occupancy was less than 0.1 s were excluded. Unit firing rate maps were smoothed using a 2-bin radius Gaussian filter. For the analysis of rate maps between two different sessions, we only used pixels in which the criterion for occupancy was met for both sessions.

We used the following metrics to compare the spatial properties of the spike rate maps of mice during the CPP and the SAL sessions:

1. Peak firing rate was defined as the maximum number of spikes per unit time for the place cell over all the pixels during a session.
2. Mean firing rate was defined as the average number of spikes per unit time for the place cell over the pixels during a session.
3. SI was calculated by estimating the rate of information *I*(*R*|*X*) between firing rate *R* and location *X* with the formula (Skaggs et al., 1993; Cacucci et al., 2007):
  $$I(R|X)\approx \sum_{i}p({\vec{x}}_{i})f({\vec{x}}_{i})\log_{2}\text{​​}\left(\frac{f({\vec{x}}_{i})}{F}\right)$$
  in which *p*(*x*_*i*_) is the probability for the animal being at location *x*_*i*_, *f(*x*_*i*_)* is the firing rate measured at location *x*_*i*_ and *F* is the overall firing rate of the cell.
4. Place field size was calculated as the area of the firing rate map in which at least 8 contiguous pixels that shared an edge had a rate ≥10% of the peak firing rate for that unit. Moreover, this area needed to contain the peak firing rate (Mehta et al., 1997; Brontons-Mas et al., 2010). Using this criterion, we excluded additional areas that had pixels with elevated firing.
5. The center of mass (COM) was calculated as the *x* and *y* averages for the rows and columns of the rate map weighted for firing rate. The COM shift was calculated as the distance of the place field's COM between two trials, with the COM vector being the graphical representation of this shift. Thus, this metric gave an account of place field movement across trials.
6. The similarity score was defined as the Pearson's product moment correlation between all matrix elements, in this case pixels in the arena depicting firing rate, of a place field between two trials (Muller and Kubie, 1987; Kentros et al., 1998).
7. Object proximity was calculated as the distance between a place field's COM and the center point of object's location.

All analyses of the described metrics were conducted using custom-written macros in Excel 14 (Microsoft), Origin 8 (Origin Lab), and Matlab 7 (Mathworks).

### Histology

Electrode positions were determined by electrolytic lesion (0.1 mA for 10 s) at each tetrode after all behavioral sessions had been completed. One day after the lesion, mice were sacrificed and brains sectioned and processed for Nissl staining (Figure 2). Recording sites were determined by comparison of sections with standard stereotaxic coordinates (Paxinos and Franklin, 2012). Although it was not possible to isolate every single tetrode track, we were able to confirm that at least one tetrode per mouse had reached the target CA1 area. Therefore, all the results from the seven implanted mice were used for final analysis.

### Statistical analysis

Data are presented as mean ± s.e.m., mean ± *SD*, or median ± interquartile range (IQR), as indicated. For each comparison, we assessed whether the samples were normally distributed with the use of the Shapiro-Wilk-test. For normal samples, we used factorial ANOVA and Student's *t*-test (typically for two samples; one-sample *t*-tests are specified) to examine statistical significance, which was defined as *P* < 0.05. For samples that were not normally distributed, we used the Kolmogorov-Smirnov (KS) test and Mann-Whitney *U*-test. Moreover, we used the chi square-test for analysis of frequency differences between classes of place cells in the CPP and SAL groups.

## Results

### NMDAR antagonism disrupted the OPM task

We used BALB/cJ females (*n* = 33) because they showed avid object exploration (Chang and Huerta, 2012). Initially, animals were exposed to the empty chamber in order to habituate them to the context and increase the salience of objects. Each mouse had 4 familiarization sessions, for a cumulative period of 1 h, over 2 days. On the third day, mice were assigned to the CPP (*n* =13), saline control (SAL, *n* = 10) or non-injected control (NIC, *n* = 10) groups and subjected to the OPM task (Figures 1A,B), as detailed in the Methods. For T1, we found that all groups spent comparable amounts of time moving within the empty chamber (CPP, 565.1 ± 58.7; SAL, 504.7 ± 75.7; NIC, 567 ± 74.5 s; mean ± s.e.m.). Notably, this pattern of movement was maintained when objects were present in the chamber on T2 (CPP, 165.7 ± 18.7; SAL, 191.5 ± 20.3; NIC, 151.7 ± 17.1 s; mean ± s.e.m.) and T3 (CPP, 182.7 ± 18.1; SAL, 188.3 ± 25; NIC, 166 ± 21.6 s; mean ± s.e.m.). Analysis of variance of animal movement, expressed as percent of total time for each trial (Figure 1C), revealed no statistical differences between groups (*P* = 0.73, *F* = 0.32) and trials (*P* = 0.7, *F* = 0.33). For T2 and T3, we tracked the mice (Figure 1B) and measured object exploration by recording the episodes in which the animal's snout was in close proximity (<1 cm) to the object's periphery. During T2, when two objects were introduced to the arena, we arbitrarily defined one as the “left object” (LO) and the other as the “RO,” depending on their position in the chamber. We found that the CPP group spent similar amounts of time visiting both objects (LO, 41.1 ± 11.7, RO, 50.3 ± 10.7 s; mean ± s.e.m.; *P* = 0.6, *T* = 0.5, *t*-test), which was also the case for the NIC group (LO, 39.2 ± 7, RO, 37 ± 6.5 s; *P* = 0.8, *T* = 0.3, *t*-test) and the SAL group (LO, 28.8 ± 8.7, RO, 23 ± 6.6 s; *P* = 0.6, *T* = 0.5, *t*-test). During T3, when one object was moved and the other remained in a stable location, we first measured the cumulative time visiting both objects to establish whether mice remained interested in their presence inside the chamber. We observed that the pattern of visits to both objects, regardless of their position, was similar between T2 and T3 for each group (Figure 1D). Surprisingly, the SAL group had less cumulative visits than the other groups on T2 (SAL vs. CPP, *P* = 0.02, *T* = 2.4; SAL vs. NIC, *P* = 0.15, *T* = 1.5, *t*-test) and T3 (SAL vs. CPP, *P* = 0.01, *T* = 2.8; SAL vs. NIC, *P* = 0.07, *T* = 1.9, *t*-test). Conversely, the CPP and NIC groups had similar cumulative visits (T2, *P* = 0.4, *T* = 0.9; T3, *P* = 0.3, *T* = 1.0, *t*-test).

When considering the times spent visiting each object separately during T3, we found that the CPP group explored the stable object and the moved object comparably (stable, 61.7 ± 12.9, moved, 40.5 ± 9.3 s; mean ± s.e.m.; *P* = 0.2, *T* = 1.3, *t*-test) but the SAL and NIC groups displayed a bias for the moved object (Figure 1E) (SAL, stable, 22.8 ± 4.1, moved, 40.8 ± 6.3 s; NIC, stable, 35.8 ± 7.3, moved, 51.6 ± 5.5 s; mean ± s.e.m.). To increase statistical power, we merged the NIC and SAL groups into a single control pool and compared the exploration ratios (for T2) and OPM ratios (for T3) against the CPP mice. We found that both groups had similar exploration ratios (CPP, 0.09 ± 0.19; control, −0.01 ± 0.09; mean ± s.e.m.; *P* = 0.6, *T* = 0.5, *t*-test), but the CPP group showed significantly lower OPM ratio (Figure 1F) (CPP, −0.2 ± 0.16; control, 0.27 ± 0.07; mean ± s.e.m.; *P* = 6 × 10^−3^, *T* = 2.9, *t*-test). Importantly, within-group comparisons revealed that for control mice the OPM ratio was higher than the exploration ratio (*P* = 0.02, *T* = 2.3, *t*-test), whereas these two ratios were not statistically different in CPP mice (*P* = 0.3, *T* = 1.2, *t*-test). Taken together, these results demonstrated that antagonism of NMDAR function by CPP interfered with appropriate performance during the OPM task.

The effect of different intervals between T2 and T3 has already been examined in mice (Murai et al., 2007), but not in the BALB/cJ strain. We thus performed the OPM task with long delays (1, 2, or 24 h). We found that the CPP group had significantly lower OPM ratios than the SAL group for 1-h and 2-h delays (1 h, CPP, *n* = 8, −0.14 ± 0.13; SAL, *n* = 8, 0.21 ± 0.07; *P* = 0.03, *T* = 2.3; 2 h, CPP, *n* = 8, −0.09 ± 0.1; SAL, *n* = 8, 0.25 ± 0.07; *P* = 0.02, *T* = 2.7; mean ± s.e.m., *t*-test) (Figure 1G). Three days later, we tested the CPP group (without injection, using a 10-min delay) which showed a robust OPM ratio (*n* = 12, 0.27 ± 0.06), demonstrating that animals recovered quickly from NMDAR antagonism. Finally, 9 days later, we retested the mice with a 24-h delay and both groups had OPM ratios at chance level (CPP, *n* = 12, 0.05 ± 0.1; SAL, *n* = 12, −0.04 ± 0.09; *P* = 0.5, *T* = 0.7; mean ± s.e.m., *t*-test) (Figure 1G). These data confirmed the published report stating that mice could perform the OPM task with intervals of few hours but not 24 h (Murai et al., 2007).

We next examined the effect of post-training CPP injections by using a T2–T3 delay of 2 h. One squad was injected with CPP (or SAL) 5 min after T2, and T3 occurred 115 min later. In this case, both groups displayed OPM ratios significantly above chance (CPP, *n* = 8,0.24 ± 0.12; SAL, *n* = 8, 0.28 ± 0.12). Another squad was injected with CPP (or SAL) 65 min after T2, with T3 occurring 55 min later. Again, both groups had strong OPM ratios (CPP, *n* = 8, 0.26 ± 0.15; SAL, *n* = 8, 0.22 ± 0.14) (Figure 1H). These data demonstrate that NMDAR antagonism after the sample but before the choice phase (with a delay of 2 h) does not alter OPM, as it was previously shown in rats (Larkin et al., 2008; Warburton et al., 2013).

### CA1 recordings in implanted mice

Mice (*n* = 7) were implanted with moveable arrays carrying tetrodes that were targeted to dorsal CA1. Following 3 d of recovery from surgery, they were thoroughly habituated to the arena over 3–5 days to increase object salience and render the hippocampal representation of the environment both robust and high in SI content. We recorded neural signals during these sessions as the array was advanced daily until CA1's *stratum pyramidale* was reached (Figure 2). Two LEDs attached to the headstage above the animal's head were used to track exploration of the arena and objects during testing (Figure 3A). The presence of electrophysiological markers, such as theta rhythm when the animal was moving (Figure 3B), and sharp waves and ripples during stationary periods, ensured that the electrode tips were in CA1. Single units with high spike amplitude were evident in most recordings and were classified off-line as putative pyramidal neurons and interneurons with standard clustering and autocorrelogram methods (Figure 3C). Pyramidal neurons were analyzed further for their place cell activity (Figure 3D). During days of testing, mice were injected with either CPP or SAL and, 30 min later, were subjected to the OPM task. Each animal experienced 1–3 CCP and 0–3 SAL sessions. We found that implanted mice, in either the CCP or SAL conditions, explored the objects as avidly as mice without implants. Predictably, CPP-injected animals did not visit the moved object preferentially during T3 (OPM ratio, 0.02 ± 0.09, mean ± s.e.m.), but displayed increased exploration of objects in T2 compared to T3 (T2, 56.3 ± 6.5, T3, 34.7 ± 2.7 visits; mean ± s.e.m.), indicating that their lack of discrimination in T3 was not due to oversampling of the objects during the last phase of the task.

### Active and inactive units during T1

**Figure 4:**
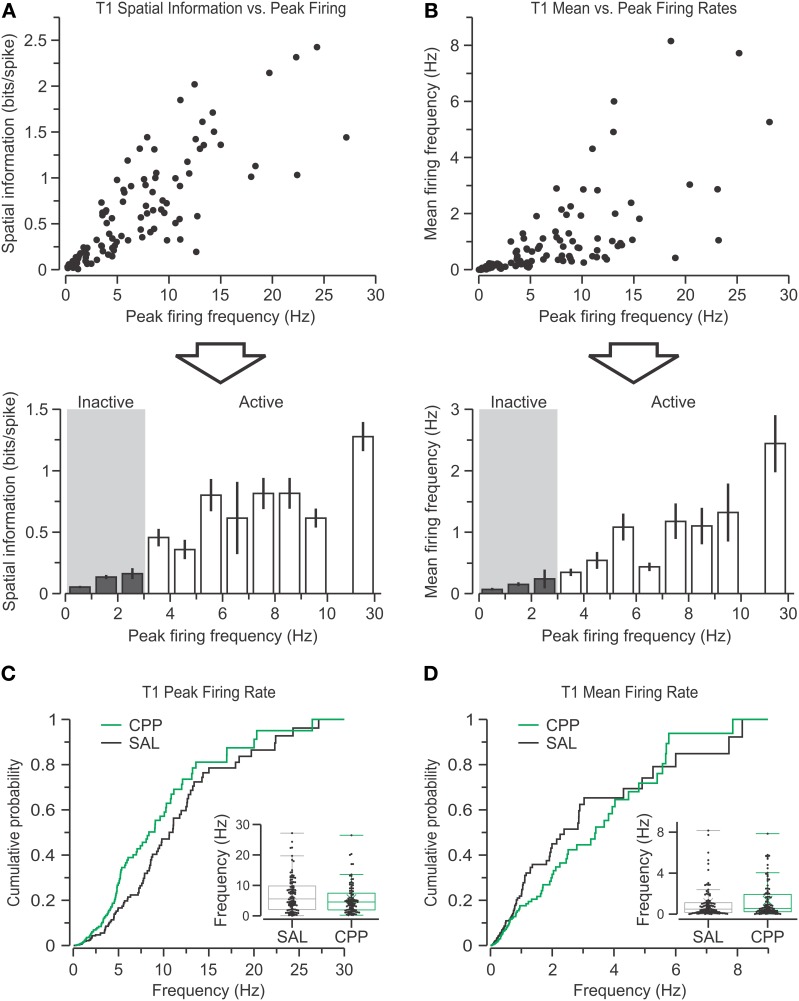
**Definition of active and inactive single units. (A)** Top, scatter plot showing spatial information vs. peak firing rate for all putative pyramidal cells from the SAL group recorded in T1 (*n* = 103). Bottom, bar graph (mean ± s.e.m.) with the data binned into 1-Hz groups (with an average of 7.8 units per bin) shows the boundary between inactive (gray area) and active units. Notice that cells with peak firing rate >10 Hz are grouped into a single bin. **(B)** Top, scatter plot of mean firing rate vs. peak firing rate of all SAL units in T1. Bottom, bar graph (mean ± s.e.m.) with the binned data also reveals the segregation of inactive (gray area) and active cells. All inactive units (except one) have a mean firing rate >1 Hz. **(C)** Peak firing rates for CPP (*n* = 97) and SAL units during T1, plotted as distributions of cumulative probabilities and box plots (inset), reveal no significant differences between groups. **(D)** Mean firing rates are also comparable among groups.

We used the activity patterns of putative pyramidal cells from SAL-injected mice during T1 to categorize them. We noticed that there was a strong correlation between peak firing rates and SI, such that units with peak firing rates <3 Hz displayed markedly low SI. Using this natural boundary, units with peak firing rates <3 Hz were categorized as *inactive units*, and those with peak firing rates ≥3 Hz were defined as *active units* (Figure 4A) (inactive, 0.082 ± 0.082; active, 0.724 ± 0.721 bits per spike; median ± IQR; *P* = 0, *D* = 0.87, *Z* = 0.19, two-sample KS test). We did not assume we had sampled all the possible inactive units in the neighborhood of each tetrode, only those that produced enough spikes during a recording session to be isolated by the clustering software. Additionally, we found a strong correlation between the unit's peak firing rates and their mean firing rates, which also aligned with the 3-Hz boundary (Figure 4B) (inactive, 0.091 ± 0.116; active, 0.789 ± 1.412 bits per spike; median ± IQR; *P* = 0, *D* = 0.88, *Z* = 0.19, two-sample KS test).

We compared the firing rates for all the units from CPP-injected sessions (*n* = 97) with those from SAL-injected sessions (*n* = 103). Since the firing rates were not normally distributed, we used the non-parametric KS test to check for statistical difference and found that the CPP group had a tendency to differ from the SAL group, but did not reach statistical significance (Figure 4C) (*P* = 0.05, *D* = 0.19, *Z* = 0.03, two-sample KS test). Moreover, the unit's mean firing rates were not significantly different between groups (Figure 4D) (*P* = 0.37, *D* = 0.13, *Z* = 0.02, two-sample KS test). In short, the silencing of NMDARs by CPP did not alter the rates of spiking in CA1 pyramidal cells.

### Classes of place cells during the OPM task

**Figure 5:**
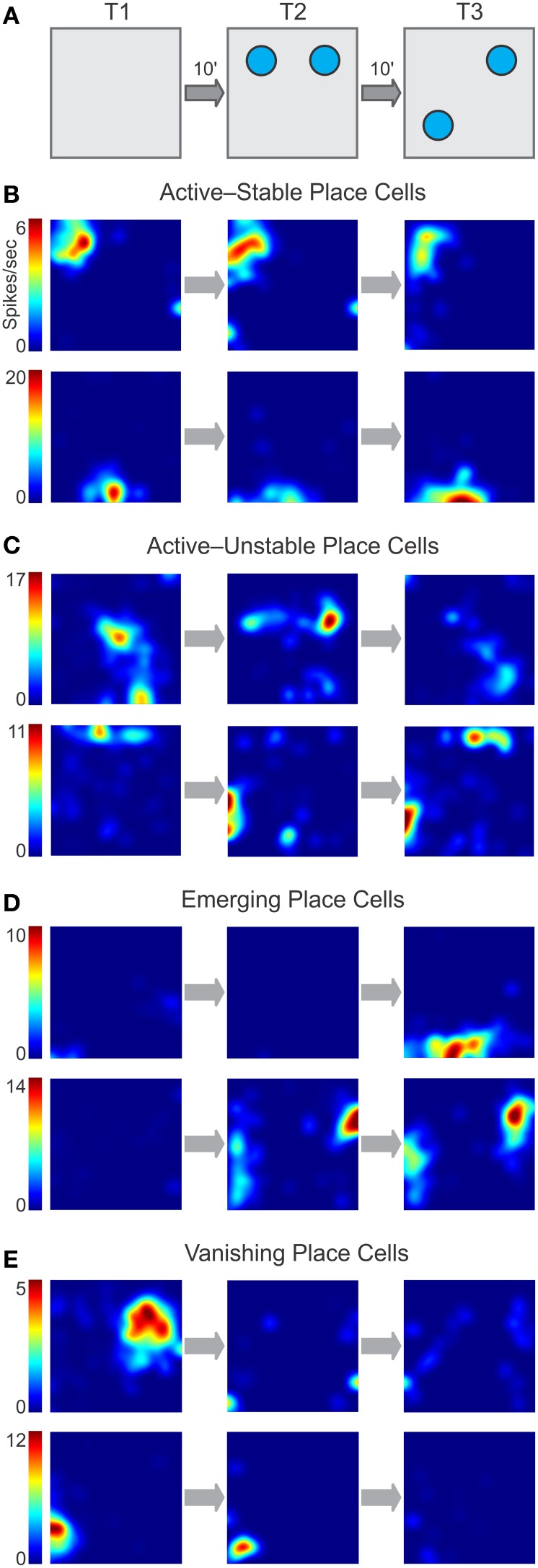
**Classes of place cell dynamics during the OPM task. (A)** Schematic of the task with the phases of familiarization (T1), exposure to two objects (T2), and object movement (T3) separated by delays of 10 min (10′). The firing rate maps in **(B–D)** correspond to these phases. **(B–C)** Active units have a peak firing rate ≥3 Hz in two consecutive phases of the task. **(B)** Some active units exhibit high stability throughout the task. **(C)** Other active units remain active throughout the task but show little stability. **(D)** Emerging units are inactive at earlier phases of the task but upon object introduction or movement display place fields *de novo*. **(E)** Vanishing units have established place fields earlier in the task but become inactive following object introduction or movement.

We observed several salient patterns of place cell activity across the OPM task (Figure 5). Most single units displayed robust place fields (with peak firing rate ≥3 Hz) in two or more consecutive phases of the task, and were thus categorized as *active* place cells. Within this class, there were units that exhibited constant place fields that did not vary during the task (Figure 5B) and were subcategorized as *stable* place cells. Other active units showed remapping in the form of place field movement, emergence of additional sub-fields, or disappearance of subfields, and were subcategorized as *unstable* place cells (Figure 5C). A minority of the units was inactive in T1 or T2 but following either object introduction or object movement developed robust place fields, and were termed *emerging* place cells (Figure 5D). Yet another set of units were active in earlier phases of the task but showed disappearing place fields, thus becoming inactive units by the end of the task, and were called *vanishing* place cells (Figure 5E). Parenthetically, in separate experiments in which mice underwent duplicate T2 sessions (with objects present, but not moved), the emerging and vanishing classes were not apparent during the second T2, with most cells falling into the stable class. Although we did not use this particular dataset for further analysis, it suggested to us that the place cell classes we isolated were associated to particular phases of the OPM task, instead of being a more general, non-specific drift of place cells.

### Active place cells were altered by CPP during T1

**Figure 6:**
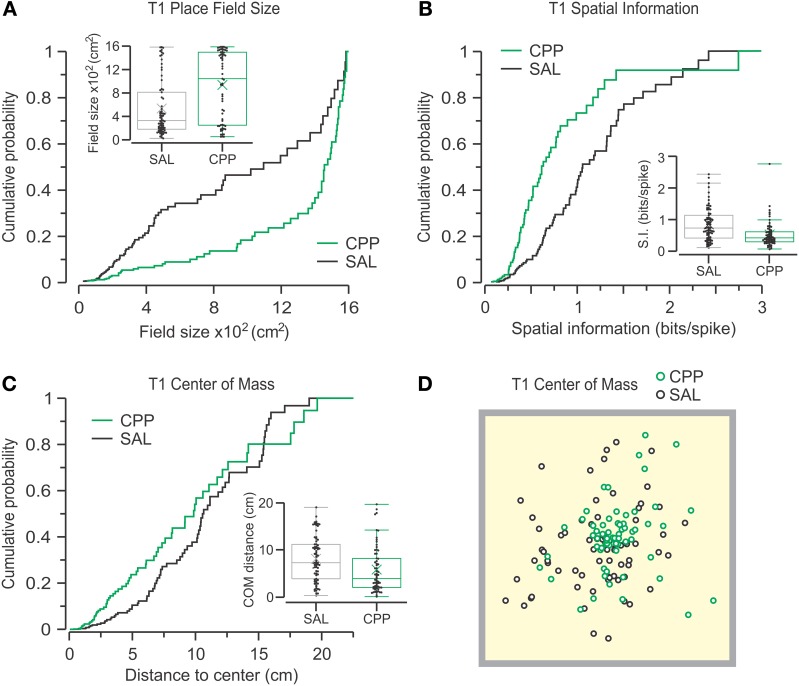
**CPP alters place cell properties during T1.** Cumulative probability distributions and box plots (insets) are used to display the parameters under analysis for active CPP (*n* = 64) and SAL (*n* = 73) units. **(A)** Place fields are defined as contiguous areas >0.1 of peak firing rate that contain the peak firing rate (no additional fields included). Place field sizes are highly different between groups (*P* = 9.2 × 10^−5^, KS test). **(B)** Spatial information is significantly different between groups (*P* = 1.3 × 10^−4^, KS test). **(C)** The distance between the place field's center-of-mass and the arena center also shows significant difference (*P* = 3.4 × 10^−3^, KS test). **(D)** Top view of the behavioral arena (40 cm on the side) with the centers-of-mass for all active units distributed throughout the chamber.

We examined whether any of the defining properties of active place cells was affected by NMDAR antagonism during T1. We observed that units from CPP-injected sessions (CPP–PCs) had much larger place field sizes than those from SAL-injected sessions (SAL–PCs) (CPP, *n* = 64, 1084 ± 1284; SAL, *n* = 73, 328 ± 632 cm^2^; median ± IQR). Statistical comparison confirmed that the place field sizes were significantly larger for CPP–PCs (Figure 6A) (*P* = 9.2 × 10^−5^, *D* = 0.37, *Z* = 0.06, two-sample KS test). Furthermore, CPP–PCs showed lowered SI as compared to SAL–PCs (CPP, 0.42 ± 0.32; SAL, 0.72 ± 0.72 bits per spike; median ± IQR), a reduction that was statistically significant (Figure 6B) (*P* = 1.3 × 10^−4^, *D* = 0.37, *Z* = 0.06, two-sample KS test). Also, CPP–PCs had the COM of their place fields more clustered around the center of the chamber (CPP, 4.03 ± 6.67; SAL, 7.25 ± 7.3 cm; median ± IQR) and this parameter was significantly different between groups (Figures 6C,D) (*P* = 3.4 × 10^−3^, *D* = 0.3, *Z* = 0.05, two-sample KS test).

### Place cell dynamic properties during T2

**Figure 7:**
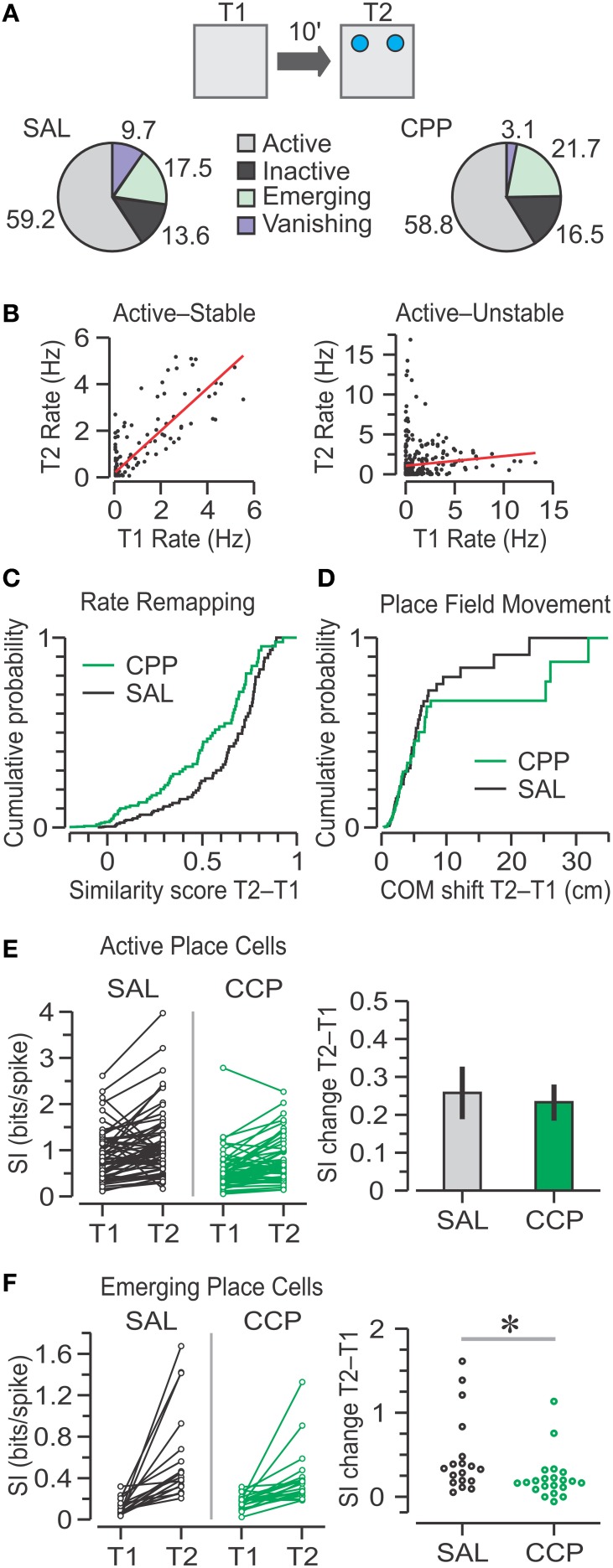
**Place cell responses to object introduction in T2. (A)** Top, schematic of the T1–T2 transition in the OPM task, with objects represented by blue circles. Bottom, pie charts with classes of units based on the activity between T1 and T2. Numbers represent the percentages of SAL (*n* = 103) and CPP (*n* = 97) units. The groups show no significant frequency differences (chi square-test). **(B)** Graphs of T2 vs. T1 mean firing rates for two active place cells, matched for all pixels occupied in both sessions. The red lines indicate linear regressions, which define similarity scores. The cell at left is stable because it has high correlation (*r* = 0.85), whereas the cell at right is unstable due to its low correlation (*r* = 0.09). **(C)** Distributions of similarity scores for active, emerging, and vanishing units for SAL (*n* = 88) and CPP (*n* = 81) groups show no statistical difference (*P* = 0.3, KS test). **(D)** Distributions of center-of-mass (COM) shifts in active units for SAL (*n* = 61) and CPP (*n* = 57) groups are not statistically different (*P* = 0.54, KS test). **(E)** Left, spatial information (SI) for active place cells in T1 and T2. Right, bar graph (mean ± s.e.m.) with SI change for active units indicating that both groups have significant increase above baseline (*P* < 0.001, one-sample *t*-tests), without statistical differences between groups (*P* = 0.76, *t*-test). **(F)** Left, SI for emerging place cells in T1 and T2. Right, SAL units (*n* = 18) undergo greater increase in SI than CPP units (*n* = 21). ^*^, *P* < 0.005 (Mann-Whitney test).

For the transition from T1 to T2, out of the four classes of single units (Figure 7A), we found that the largest percentage was active place cells (CPP, 59%; SAL, 59%) followed by emerging place cells (CPP, 22%; SAL, 18%). Moreover, we found no significant frequency differences in any of the categories between SAL and CPP groups following object introduction in T2 (χ^2^ = 4.09, *C* = 8, chi square-test).

We determined the extent of rate remapping from T1 to T2 by calculating a *similarity score* for the population of place cells, excluding inactive units. The similarity score provided an inclusive method for quantifying rate remapping between two sessions, without making a distinction whether place cells were stable or unstable (Figure 7B). We did not observe significant differences in similarity scores between groups (Figure 7C) (CPP, *n* = 81, 0.38 ± 0.61; SAL, *n* = 88, 0.49 ± 0.60; median ± IQR; *P* = 0.3, *D* = 0.14, *Z* = 0.02, two-sample KS test).

Since the similarity scores did not reflect the degree of place field movement, for active place cells we examined a parameter termed *COM shift*, which corresponded to the distance between the COM of the place field in T1 and the COM of the place field in T2. Most active CPP–PCs and SAL–PCs showed small shifts after object introduction, but a minority underwent large field movement. Nevertheless, there was no significant difference in COM shift between groups (Figure 7D) (CPP, *n* = 57, 2.76 ± 3.19; SAL, *n* = 61, 3.02 ± 3.34 cm; median ± IQR; *P* = 0.5, *D* = 0.14, *Z* = 0.03, two-sample KS test). This result contrasted with the initial differences in place field's COM distribution in T1 (Figures 5C,D).

When rodents are introduced to a novel environment, CA1 place cells are known to display an increase in SI that is thought to require NMDAR-mediated plasticity (Cacucci et al., 2007). Therefore, we addressed whether a comparable SI enhancement occurred following the introduction of two novel objects into a familiar environment. Indeed, upon presentation of objects in T2, active place cells from both groups exhibited large increases in SI. Within-group comparisons of SI increases from T1 against T2 values yielded highly significant differences for CPP–PCs (*n* = 57, 0.23 ± 0.05 bits per spike, mean ± s.e.m.; *P* = 1 × 10^−5^, *T* = 4.86, one-sample *t*-test) and SAL–PCs (*n* = 61, 0.26 ± 0.07 bits per spike, mean ± s.e.m.; *P* = 4.5 × 10^−4^, *T* = 3.7, one-sample *t*-test). However, a comparison between the CPP and SAL groups revealed no differences in SI change on T2 (Figure 7E) (*P* = 0.8, *T* = 0.3, *t*-test), a result that was not in line with the significant SI differences between groups observed during T1 (Figure 5B).

Emerging place cells represented a particularly interesting case in terms of SI. Since this category comprised a minority of the total population, we thought they might play a crucial role in encoding object location, and that this property would be reflected in large increases in SI from a low baseline content. Indeed, emerging place cells from both groups exhibited non-normal distributions of modest to large enhancements in SI, yet the SI change in CPP–PCs (*n* = 21, 0.17 ± 0.11 bits per spike, median ± IQR) was lower than the SI change in SAL–PCs (*n* = 18, 0.33 ± 0.29 bits per spike, median ± IQR), a reduction that was statistically significant (Figure 7F) (*P* = 0.01, *U* = 101, *Z* = 2.47, summed ranks = 488, Mann-Whitney-test). The number of vanishing CPP–PCs was too small to compare with SAL–PCs, although those from the SAL group appeared to exhibit a large decrease in SI (data not shown).

### Effect of object proximity on the center of mass of place cells

**Figure 8:**
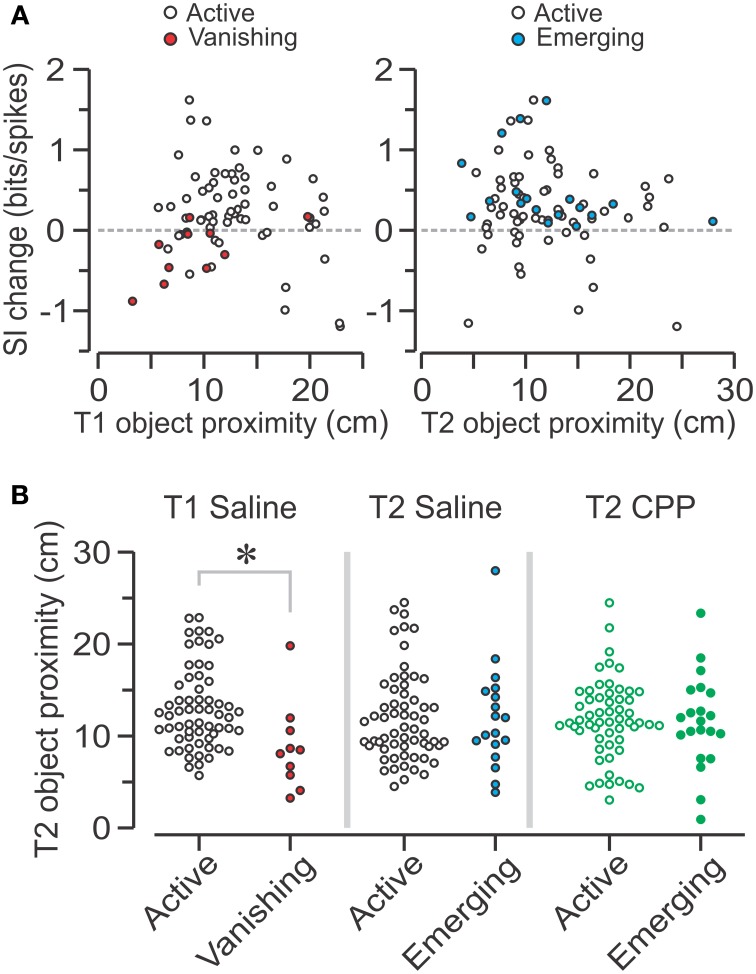
**Effect of object proximity on place cells in T2. (A)** Left, scatter plot showing the spatial information (SI) change from T1 to T2 vs. T1 object proximity, defined as the distance between the field's COM in T1 and the center of the object nearest to such place field in T2, for active and vanishing SAL units. Right, scatter plot showing SI change vs. T2 object proximity (distance between the field's COM in T2 and the center of the nearest object in T2) for active and emerging SAL units. **(B)** Left, graph showing T2 object proximity for SAL cells which are either active or vanishing in T1. The latter are significantly closer to object location than active units (^*^, *P* < 0.006, Mann-Whitney test). Middle, T2 object proximity for SAL cells that are either active or emerging in T2 shows no significant difference between groups (*P* = 0.9, Mann-Whitney test). Right, no differences in object proximity are observed for active and emerging CPP units (*P* = 0.85, *t*-test).

Considering the variety of changes in spatial coding following object introduction, we asked whether any of these modifications occurred as a function of *object proximity*, which was defined as the distance between the place field's COM (either in T1 or T2) and the center of the object nearest to such place field in T2. We first analyzed similarity scores and found that there was no significant correlation between these scores and object proximity for either group in T1 or T2 (data not shown). With regard to SI change, although there appeared to be trends in the SAL group, no significant correlations were observed between SI change and object proximity in T1 and T2 (Figure 8A). Notably, when comparing active SAL–PCs with vanishing SAL–PCs during T1, we found that vanishing units were significantly closer to the future object location (Figure 8B, left) (active, *n* = 61, 12.34 ± 5.65, summed ranks = 2363; vanishing, *n* = 10, 8.57 ± 4.34 cm; summed ranks = 193; median ± IQR; *P* = 6 × 10^−3^, *U* = 138, *Z* = 2.75, Mann-Whitney-test). There were not enough vanishing CPP–PCs in T1 to perform a similar analysis.

For T2, we compared the shortest distance to either object for active and emerging units. We observed no differences in object proximity for SAL–PCs (Figure 8B, middle) (active, *n* = 61, 10.77 ± 5.71, summed ranks = 2429; emerging, *n* = 18, 11.17 ± 5.76 cm, summed ranks =731; median ± IQR; *P* = 0.9, *U* = 538, *Z* = 0.12, Mann-Whitney-test). Moreover, no differences in object proximity were observed for active and emerging CPP–PCs (Figure 8B, right) (active, *n* = 57, 11.80 ± 0.57; emerging, *n* = 21, 5.03 ± 1.10 cm; mean ± s.e.m.; *P* = 0.85, *T* = 0.19, *t*-test).

### Place cell dynamic properties during T3

**Figure 9:**
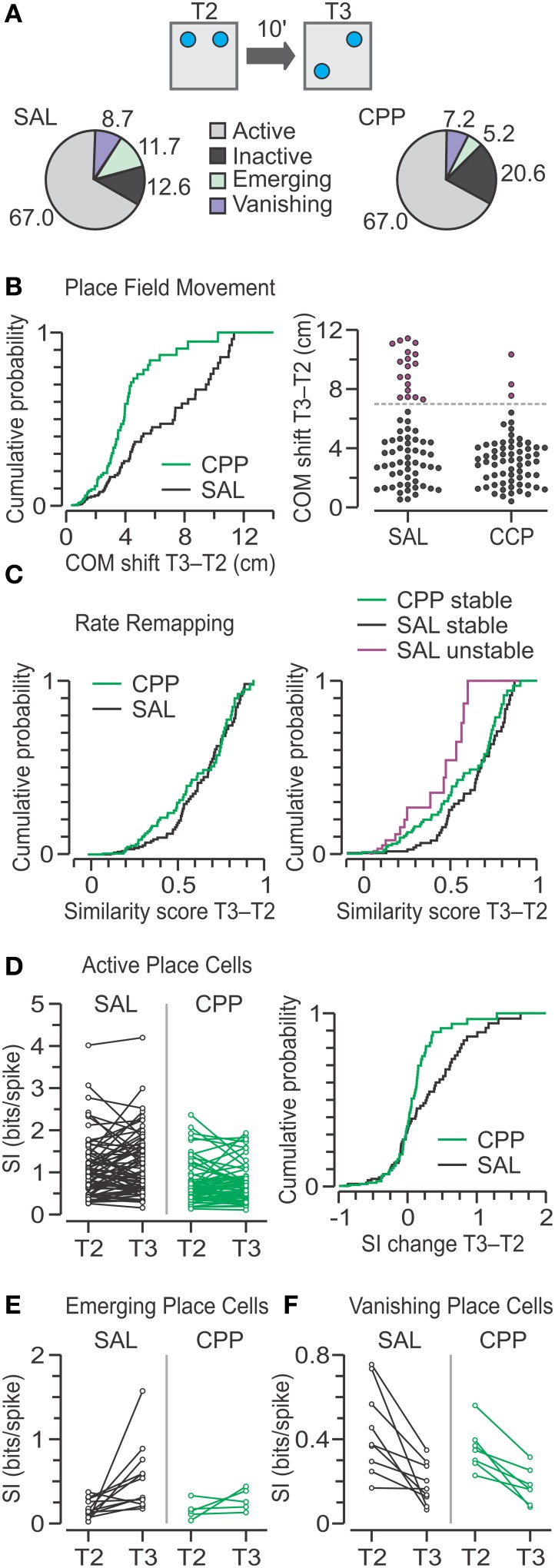
**Place cell responses to object movement in T3. (A)** Top, schematic of the T2–T3 transition in the OPM task, with objects represented by blue circles. Bottom, pie charts with classes of units based on the activity between T2 and T3. Numbers represent the percentage from total for SAL (*n* = 103) and CPP (*n* = 97) units. There are no significant frequency differences between groups (chi square-test). **(B)** Left, distributions of COM shifts in active place cells for SAL (*n* = 69) and CPP (*n* = 65) groups are significantly different (*P* < 0.005, KS test). Right, agglomerative clustering of COM shifts yields two subsets, stable (black circles) and unstable (purple circles) units, for both the SAL and CPP groups, which are separated by the dashed line. When grouped in this manner, there are significant frequency differences between groups (*P* < 0.005, chi square-test). **(C)** Left, distributions of similarity scores for active SAL and CPP groups show no statistical difference (*P* = 0.23, KS test). Right, distributions of similarity scores, segregated into stable and unstable classes, reveal a large difference between stable (*n* = 51) and unstable (*n* = 18) SAL cells (*P* < 0.001, KS test). Moreover, stable CPP cells (*n* = 62) are mildly different than stable SAL cells (*P* < 0.02, KS test). **(D)** Left, spatial information (SI) for active place cells in T2 and T3. Right, distributions of SI change for active units with a slightly lower SI change for CPP cells (*P* < 0.04, KS test). **(E)** SI for emerging place cells in the SAL (*n* = 12) and CPP groups (*n* = 5) in T2 and T3. **(F)** SI for vanishing place cells in the SAL (*n* = 9) and CPP groups (*n* = 7) in T2 and T3.

For the transition from T2 to T3, when one object was moved, we compared the classes for CPP–PCs (*n* = 97) and SAL–PCs (*n* = 103) and found that the largest class was that of active place cells (CPP, 67%; SAL, 67%) followed by inactive units (CPP, 21%; SAL, 13%) (Figure 9A). Furthermore, there were no significant frequency differences in the active, inactive, emerging and vanishing categories between SAL–PCs and CPP–PCs (χ^2^ = 4.57, *C* = 8, chi square-test). However, there were fewer emerging place cells during T3, for both groups, as compared to T2.

For the active place cells, we analyzed the movement of their fields with the COM shift metric. While active CPP–PCs had only modest COM shifts, their counterparts in the SAL group showed a widespread range of values (Figure 9B). Indeed, a statistical comparison revealed significant differences between groups (CPP, *n* = 65, 3.06 ± 2.29; SAL, *n* = 69, 3.77 ± 5.02 cm; median ± IQR; *P* = 4 × 10^−3^, *D* = 0.3, *Z* = 0.05, two-sample KS test). Moreover, we noted that active SAL–PCs could be naturally subdivided into a subset that experienced modest COM shifts (<7 cm) and corresponded to stable place cells (*n* = 51), and a second subset that underwent large COM shifts (≥7 cm) and consisted of unstable place cells (*n* = 18) (Figure 9B, right). The latter likely contributed maximally to the difference between CPP and SAL groups. When grouped in this manner there were significant frequency differences between active CPP–PCs and SAL–PCs (χ^2^ = 11.68, C = 4, chi square-test).

The similarity scores for active place cells were not different between groups (Figure 9C, left) (CPP, *n* = 65, 0.42 ± 0.51; SAL, *n* = 69, 0.51 ± 0.36; median ± IQR; *P* = 0.23, *D* = 0.17, *Z* = 0.03, two-sample KS test). A comparison between stable and unstable SAL–PCs revealed that the former exhibited significantly greater similarity scores than the latter (Figure 9C, right) (stable, *n* = 51, 0.61 ± 0.32; unstable, *n* = 18, 0.18 ± 0.46; median ± IQR; *P* = 9 × 10^−4^, *Z* = 0.14, *D* = 0.5, two-sample KS test). Moreover, a between-group comparison revealed that stable CPP–PCs had significantly lower between-trial similarity compared to stable SAL–PCs (CPP, *n* = 62, 0.45 ± 0.52; SAL, *n* = 51, 0.61 ± 0.32, median ± IQR; *P* = 0.01, *D* = 0.26, *Z* = 0.05, two-sample KS test).

Analysis of SI change demonstrated that active CPP–PCs had significantly less SI increase than active SAL–PCs (Figure 9D) (CPP, *n* = 65, 0.02 ± 0.29; SAL, *n* = 69, 0.01 ± 0.65 bits per spike; median ± IQR; *P* = 0.03, *D* = 0.24, *Z* = 0.04, two-sample KS test). For emerging place cells, the SI changes appeared comparable to the differences observed in T2 but probably due to low numbers, no statistical differences were observed between groups during T3 (Figure 9E) (CPP, *n* = 5, 0.03 ± 0.22, summed ranks = 34; SAL, *n* = 12, 0.21 ± 0.48 bits per spike, summed ranks = 119; median ± IQR, *P* = 0.27, *U* = 19, *Z* = 1.1, Mann-Whitney-test). Finally, for vanishing place cells, there were no detectable differences in SI change between the CPP and SAL groups (Figure 9F) (CPP, *n* = 7, −0.18 ± 0.03; SAL, *n* = 9, −0.25 ± 0.07; mean ± s.e.m.; *P* = 0.35, *T* = 0.98, *t*-test).

**Figure 10:**
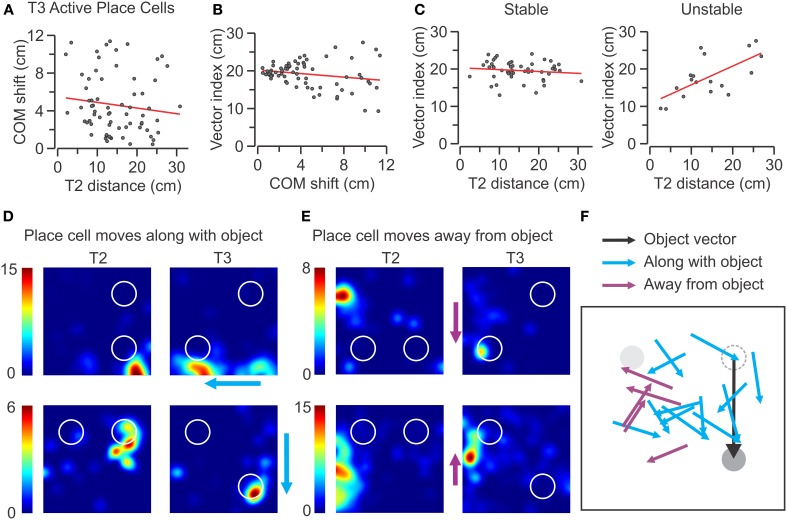
**Effect of object proximity on COM shift of place fields in T3. (A–C)** Scatter plots for active place cells from the SAL group recorded in T3 (*n* = 69). The red lines indicate linear regressions. **(A)** COM shift vs. T2 distance to the moving object for active SAL cells shows no correlation between terms (*r* = −0.03). **(B)** Vector index vs. COM shift shows negligible correlation (*r* = −0.17). **(C)** Active SAL cells are segregated into stable (left) and unstable (right) classes. No significant correlation is observed for stable units (*n* = 51, *r* = −0.07), whereas unstable units have a significantly positive correlation between vector difference and T2 distance to moving object (*n* = 18, *r* = 0.64, *P* < 0.01). **(D)** Representative rate maps of unstable place cells that follow the movement of the object during T3. The color scale (at left of each map) indicates the firing rate in Hz. **(E)** Rate maps for place cells that move away from the moving object during T3. Color scale as in **(D)**. **(F)** Diagrammatic representation of place cells that move along with the object and cells that move away from it. In the diagram, the objects (in T2) are aligned to the NW (gray), and NE (dashed) sectors, although they were tested in all the quadrants. The moving object (in T3) is represented along the object vector (black line).

The tantalizing observation that many SAL–PCs displayed large movements of their place fields was pursued further by asking whether the COM shifts occurred in a stereotyped manner based on the location and direction of the moving object. We first investigated whether place cells with place fields near the moving object showed the largest field changes on T3. When plotting the COM shifts of all the active SAL–PCs against T2 distance (the length between the COM of the place field and the center of the moving object), we found no correlation between the terms (Figure 10A) (*n* = 69, *r* = −0.03). Thus, the units closer to the moving object in T2 did not have a tendency to show a higher COM shift on T3. Next, we defined a *vector index* as the difference between the *moving object vector* (20 cm, the length the object was displaced between T2 and T3) and the *COM vector* (the distance of the COM of the place field between T2 and T3), where a null vector index indicated a place field moving along the same vector as the object. We found that, for all the active SAL–PCs, there was a somewhat negative correlation between the vector index and COM shift on T3 (Figure 10B) (*n* = 69, *r* = −0.17). Remarkably, when we analyzed the stable and unstable subsets separately, we observed that the vector index was significantly correlated with T2 distance for the unstable units (*n* = 18, *r* = 0.64, *P* < 0.01) (Figure 10C), indicating that for these cells, those initially close to the moving object were more likely to move along with the object (Figures 10D,F) and those initially far from the object were more likely to move against the direction of the object (Figures 10E,F). We also found that there was a null correlation between the vector index and T2 distance for the stable units (*n* = 51, *r* = −0.07) (Figure 10C), demonstrating that object movement did not alter them.

## Discussion

We have studied dynamic properties of CA1 place cells during the OPM task that may participate in encoding OPM through NMDAR-dependent mechanisms. By antagonizing NMDAR activity with systemically injected CPP, we observe that place cells have larger fields and lower information content than in sessions in which mice receive SAL injections. On T2, when two objects are introduced to a familiar chamber, previously inactive place cells (with fields emerging *de novo* on T2) exhibit robust increases in information content, a property that is largely blunted by NMDAR antagonism. On T3, when an object is moved, CPP–PCs show lower SI increase and reduced place field movement than Sal–PCs.

The neural substrate for OPM has been only partially elucidated, but based on ablation and pharmacological studies, the postsubiculum and CA1 appear to be critically involved. This makes it likely that other highly connected regions, such as the medial entorhinal cortex, dentate gyrus, CA3, and subiculum, participate in OPM. Our approach of peripherally injecting CPP is obviously not ideal for isolating which regional NMDAR-mediated processes are essential for OPM. Nevertheless, an examination of the pattern of connectivity between these areas indicates that CA1 is an optimal region to study, because it represents an obligatory output of the circuit. In addition, the firing patterns of CA1 place cells in response to object manipulations have been previously characterized (Lenck-Santini et al., 2005; Komorowski et al., 2009; Manns and Eichenbaum, 2009; Burke et al., 2011; Deshmukh and Knierim, 2013).

The results during T1, in which CPP–PCs show enlarged place fields and low SI show a striking resemblance with published data from relevant mutant strains, such as hippocampus-restricted NR1-KO mice (McHugh et al., 1996), transgenic mice with a T286A point mutation in the α-isoform of the calcium-calmodulin-dependent protein kinase II (Cacucci et al., 2007), parvalbumin (PV)-specific NR1-KO mice (Korotkova et al., 2010), and GluA1-KO mice (Resnik et al., 2012). We favor the possibility that CPP interrupts the NMDAR-dependent nonlinear integration of postsynaptic responses in excitatory neurons (Gasparini et al., 2004; Polsky et al., 2004), which we believe would have direct consequences for within–field burst firing and information content. This integrative mechanism seems more plausible than a CPP blockade of NMDAR-dependent synaptic plasticity on pyramidal cells because, during T1, the mouse is exposed to a highly familiar chamber, making it unlikely that NMDAR-dependent synaptic plasticity is required for maintaining an already highly stable CA1 spatial code. Concerning disruptions on NMDAR-expressing interneurons, CPP most likely interferes with network coordination mediated by interneurons that express NMDARs (Korotkova et al., 2010). For instance, if one assumes partial or total inhibition of NMDARs in PV-positive interneurons, our results during T1 would recapitulate the place field phenotype of PV-NR1-KO mice, independent of any CPP effect on pyramidal cells or other interneuron classes. Indeed, considering that PV-NR1-KO mice are impaired in a task that is somewhat similar to the one we used in this study, the deleterious effects of CPP over the OPM task may be partly explained by an effect on PV-positive interneurons. However, a primary role of these cells is to coordinate temporal coding of hippocampal pyramidal cells (Korotkova et al., 2010; Royer et al., 2012). Thus, we do not have an *a priori* reason to assume that their dysfunction would interfere with the changes in rate coding of CA1 pyramidal cells that we observe under CPP influence.

The increased field size and decreased SI of CPP–PCs might be responsible for the subsequent impaired performance in the OPM task by CPP-treated mice. This possibility seems unlikely, given that a recent study in rats shows that they can successfully discriminate a moved object when they are injected with CPP directly after but not prior to training in an OPM task (Larkin et al., 2008; Warburton et al., 2013). In our hands, we also did not observe any effect of CPP when applied in post-training interval.

The results during T2 reveal that active CPP–PCs and SAL–PCs do not differ in their values for similarity scores, COM shifts and SI changes, implying that certain dynamic properties of already established place cells do not require NMDAR function to encode a local disturbance of the environment, such as the introduction of objects in the arena. Nevertheless, active place cells from both groups show clear perturbations during T2. In particular, the low values for similarity scores and the high values for COM shifts demonstrate that the CA1 spatial representation is sensitive to the introduction of objects, as reported previously (Burke et al., 2011; Deshmukh and Knierim, 2013). A novel finding of this study is that the SI of place cells in T2 increases significantly higher than baseline values in T1 in an NMDAR-independent manner. This suggests that the information content of place cells increases as a function of the enrichment of environmental cues, in this case objects, in a previously cue-poor square environment (Hetherington and Shapiro, 1997).

Remarkably, about 20% of the units recorded during T2 correspond to emerging place cells, in both the CPP and SAL groups. It is possible that this substantial number of cells may be a byproduct of cue enrichment or, alternatively, that it encompasses a unique subset that is engaged in the representation of objects and space. The latter view is strengthened when considering that emerging CPP–PCs exhibit significantly lower SI increase, suggesting that the information-rich emerging SAL-PCs play a key role in binding objects and space during T2. It is thus possible that emerging place cells are critical in building a spatial representation that includes the newly object-modified environment and that this representation may be required for further discrimination of object location.

The results during T3, following the movement of one object, reveal that active CPP–PCs display only minor movements in their place fields, whereas active SAL–PCs can be neatly divided into a large subset with negligible field movement and a smaller subset with extensive COM shift. Not only is the difference in the COM shift distributions highly significant between the CPP and SAL groups, but also the cluster of unstable SAL–PCs (with large COM shift) follows a topographical pattern based on the vector of the moving object.

It is likely that the increased reproducibility and information content displayed by the SAL–PCs belonging to the active and stable class during T3 obeys the same principles observed by other groups (Wilson and McNaughton, 1993; Frank et al., 2004; Cacucci et al., 2007) following exposure to novel environments. In our study, the introduction of objects during T2 may provide novel input and cause some initial remapping, but those cells in which object movement (during T3) does not produce additional remapping would be more reproducible and information-rich. Alternatively, the increased stability might be a function of attention, in which the discriminating animals are more attentive to the moved object, leading to a spatial representation that remains more similar between sessions (Kentros et al., 2004). Although the increased information content and increased stability of the majority of active place cells may not be a primary mechanism for encoding object location, it may serve a role in the discrimination of a changed environment, such as one in which an object has been moved.

Overall, our study contributes to the elucidation of the mechanisms by which CA1 ensembles encode and represent the location of objects within an environment. We propose that the stable spatial map that is generated by CA1 place cells, after numerous exposures to the same environment, can also signal the introduction of objects to the environment. Our key observation is that parameters, such as information content and movement of the place field's center, that typically describe spatial properties of place cells can also account for the response of these cells to local objects. In particular, we propose that the CA1 ensemble representation of stable and unstable place cells at the new object location constitutes a place-specific novelty signal that we think might be responsible for discrimination of the moved object by the animal. Future studies may aim to gain a firmer sense of the stability of the place code when objects, and their movement, are included in the behavioral paradigms. For instance, animals may undergo several T2 sessions before T3, which may lead to an enhancement of the place novelty signal. Another possibility would be to examine the stability of the place cell code for behavioral manipulations that include richer assortments of object manipulations (Dere et al., 2007).

A somewhat surprising finding in our study is that the introduction of objects, during T2, causes substantial place field remapping of active place cells that is independent of NMDARs. Therefore, we believe most changes in spatial coding, including place field similarity, movement, and increase in information content do not require NMDAR activation. Since we used systemic CPP, this observation applies not only to CA1 but also to its input areas. It is thus more likely that NMDAR-dependent plasticity of the new T2 pattern of synaptic inputs, which are responsible for the remapping observed, causes place cells to be allocentrically controlled by novel environmental cues when the object is moved in T3, resulting in the targeted remapping of a subset of place cells.

In conclusion, we used the OPM task that produced robust, spontaneous object exploration in mice to study whether CA1 place cells signaled the introduction or movement of objects by NMDAR-mediated changes of their spatial coding. We found that certain metrics such as SI content and place field movement (expressed as COM shifts) reflected important dynamic properties that were mediated by NMDARs and could be responsible for binding object identity with location. Our findings highlight a role for CA1 in encoding object location within the spatially related place cell activity.

### Conflict of interest statement

The authors declare that the research was conducted in the absence of any commercial or financial relationships that could be construed as a potential conflict of interest.
